# Antimicrobial Resistance in Children With Bloodstream Infections: A Population‐Based Study in Stockholm, Sweden

**DOI:** 10.1111/apa.70683

**Published:** 2026-07-14

**Authors:** Joachim Luthander, Rutger Bennet, Per Nydert, Margareta Eriksson, Christian G. Giske, Anna Nilsson

**Affiliations:** ^1^ Division of Pediatric Infectious Diseases, Astrid Lindgren Children's Hospital Karolinska University Hospital Stockholm Sweden; ^2^ Division of Clinical Pediatrics, Department of Women's and Children's Health Karolinska Institutet Stockholm Sweden; ^3^ Department of Clinical Science, Intervention and Technology Karolinska Institutet Stockholm Sweden; ^4^ Department of Laboratory Medicine, Clinical Microbiology Karolinska Institutet Stockholm Sweden; ^5^ Department of Clinical Microbiology Karolinska University Hospital Stockholm Sweden; ^6^ Childhood Cancer Research Unit, Department of Women's and Children's Health Karolinska Institutet Stockholm Sweden

**Keywords:** antimicrobial resistance, bloodstream infections, children

## Abstract

**Aim:**

We retrospectively evaluated trends in antimicrobial resistance (AMR) in paediatric bloodstream infections and assessed changes in antimicrobial prescription patterns over a 20‐year period.

**Methods:**

We analysed positive blood cultures from children admitted with suspected serious infection between 1998 and 2018. Data on comorbidities, hospital unit, intensive care admission, community‐ or hospital‐acquired isolates, and mortality were collected. Resistance results were compared with national surveillance data.

**Results:**

Among 1977 isolates, crude AMR was 9.2%, increasing in Gram‐positive (*τ* = 0.61, *p* < 0.001) and Gram‐negative bacteria (*τ* = 0.54, *p* < 0.01). ESBL‐producing Enterobacterales increased from 1.5% to 14.1% (*p* < 0.001); 64.7% were multidrug‐resistant. Most children with ESBL‐producing Enterobacterales (82.4%) or methicillin‐resistant *Staphylococcus aureus* (75%) had been hospitalized within the previous 6 months or had household members with travel to or origin from high‐AMR countries. Overall antimicrobial use remained stable, although prescriptions of third‐generation cephalosporins, piperacillin‐tazobactam, and carbapenems increased. AMR rates remained lower than in many countries and were comparable to Swedish adult data.

**Conclusion:**

AMR increased despite stable antimicrobial use. Empirical coverage for resistant pathogens should be considered in children with recent hospitalization, travel to, or household origin from regions with high AMR prevalence.

## Summary

- The prevalence of ESBL‐producing Enterobacterales is increasing; risk factors include recent hospitalization and travel history.
- Contemporary resistance data are essential for evidence‐based empirical antibiotic therapy.
- Surveillance, early detection, and targeted interventions are key to antibiotic stewardship and limiting AMR dissemination.

**Abbreviations**
AMR: antimicrobial resistance
ARPEC: Antibiotic Resistance and Prescribing in European Children
BSI: bloodstream infection
CAI: community‐acquired infections
EARS‐net: European Antimicrobial Resistance Surveillance Network
ECDC: European Centre for Disease Prevention and Control
ESBL: extended‐spectrum β‐lactamase‐producing Enterobacterales
ESBL_CARBA_
: carbapenemase‐producing ESBL
EUCAST: European Committee for Antimicrobial Susceptibility Testing
GN: gram‐negative bacteria
GP: gram‐positive bacteria
HAI: healthcare‐associated infections
MDR: multidrug resistance
MRSA: Methicillin‐resistant *Staphylococcus. aureus*
NICU: neonatal intensive care unit
PICU: paediatric Intensive Care Units
PNSP: Penicillin non‐susceptible *Streptococcus. pneumoniae*
SVEBAR: Swedish Surveillance of Antibiotic Resistance

## 1 Introduction

Bloodstream infection (BSI) contributes to childhood morbidity and mortality. Effective treatment requires early administration of empirical antimicrobial therapy. Antimicrobial resistance (AMR) poses a major threat to effective therapy and human health. The global burden of AMR is estimated to be a leading cause of death worldwide, with 1.14 million deaths attributable to AMR in 2021 [1]. However, the global decline in sepsis‐related deaths from 1990 to 2021 has reduced AMR‐related mortality by more than 50% in children under the age of 5 and coincides with widespread vaccination efforts and improvement in water, sanitation, and hygiene [1]. Six major bacterial pathogens are responsible for 83% of AMR‐related deaths, though the burden of each pathogen differs substantially across regions [1]. European data on BSI indicate that the highest burden of infections with antibiotic‐resistant bacteria occurs in infants aged < 1 year [2]. Recognizing the severity of the issue, the World Health Organization has declared AMR one of the most urgent health threats of our time and has endorsed a global action plan to mitigate the emergence and spread of resistance. Surveillance of AMR is crucial for strengthening the knowledge base that informs policy and clinical decision‐making.

Sweden has a long‐standing tradition of preventing antibiotic overuse in primary healthcare and hospitals, contributing to a comparatively low prevalence of AMR (3). However, AMR has increased over the last two decades [3]. National surveillance reports often combine data from adult and paediatric populations despite significant differences in resistance patterns between these groups, partially attributable to variations in antibiotic prescribing practices [4]. In general, AMR occurrence has been reported to be lower in children than in adults [5]. In Sweden, outpatient antibiotic sales for children aged 0–18 years have declined over recent decades, with the most substantial reduction observed among children aged 0–4 years [3]. This decline can be attributed to the implementation of national guidelines promoting restrictive antimicrobial use, as well as a lower incidence of bacterial respiratory infections following the introduction of immunization programs against *Haemophilus influenzae type B* in 1992 and *Streptococcus pneumoniae* in 2008. However, antibiotic consumption trends in hospitalized children have not mirrored those observed in outpatients. Although a reduction in the use of some antibiotic classes has been reported, overall antibiotic consumption in hospitalized children has increased [6]. Despite these trends, data on paediatric AMR and antibiotic use in hospitalized children in Sweden remain limited.

This study aimed to evaluate trends in AMR among children with BSI over a 20‐year period. Additionally, the analysis focused on changes in antimicrobial prescription patterns at our hospital during the same timeframe.

## 2 Patients and Methods

### 2.1 Case Identification

This retrospective study analysed paediatric patients aged 0–17 years with positive blood cultures collected between July 1, 1998, and July 30, 2018. Blood cultures were obtained from children admitted to Karolinska University Hospital with a clinical suspicion of serious infection, including sepsis, meningitis, pneumonia, pyelonephritis, bacterial gastroenteritis, osteomyelitis, or cellulitis requiring hospitalization and intravenous antibiotics. The Department of Clinical Microbiology Laboratory Information System at Karolinska University Hospital provided data on patient identity, bacterial species, and antimicrobial susceptibility. Only bacterial isolates were included in this study. For paediatric patients with AMR, additional data on comorbidities, hospital units, intensive care unit (ICU) admission, community‐ or hospital‐acquired infection status, and mortality were collected from hospital medical records. Data on prior hospitalizations and travel history within the previous 6 months were retrospectively obtained from patients' medical records. Comorbidities included chronic diseases, immune deficiencies, congenital malformations, or acquired morbidity conditions such as necrotizing enterocolitis (NEC) and post‐surgical complications. Based on these factors, we categorized the study population into four risk groups: (1) children without underlying comorbidities, (2) neonates in the neonatal intensive care unit (NICU), (3) children with underlying comorbidities, and (4) oncologic patients. BSI were classified as community‐acquired infections (CAI) if they occurred within 48 h of hospital admission and healthcare‐associated infections (HAI) if they developed after 48 h.

### 2.2 Antimicrobial Susceptibility Testing

Susceptibility testing was performed using the disk diffusion method following the recommendations of the Swedish Reference Group for Antibiotics until 2011 [7]. After 2011, testing followed the methodology recommended by the European Committee for Antimicrobial Susceptibility Testing (EUCAST) (www.eucast.org). No standardized European recommendations existed before 2011.

Extended‐spectrum β‐lactamase‐producing Enterobacterales (ESBL)‐detection was done with the double‐disk synergy testing (DDST)‐method as described by EUCAST (https://www.eucast.org/bacteria/important‐additional‐information/resistance‐detection/). Detection of carbapenemases was done according to EUCAST algorithms. Isolates with meropenem zone diameters < 28 mm were subjected to molecular analysis for the five most common carbapenemases, and in parallel also to phenotypic testing with Mast combination disks containing meropenem+/−beta‐lactamase inhibitors as described in EUCAST guidelines.

Coagulase‐negative staphylococci, *Viridans streptococci*, Bacillus spp., Propionibacterium spp., Corynebacterium spp., and Micrococcus spp. were considered contaminants and excluded from the analysis due to challenges in determining true pathogenicity in this retrospective study. Additionally, blood cultures from a previously included patient that were positive for the same organism within 4 weeks of the initial isolate were considered duplicates and excluded.

This study reported susceptibility testing results for isolates classified as “resistant” (R). Among Gram‐positive (GP) bacteria, *S. aureus* resistant to methicillin and lincosamides, *S. pneumoniae* resistant to macrolides and lincosamides or non‐susceptible to penicillin, *S. agalactiae* resistant to macrolides, *Enterococcus faecium* resistant to vancomycin, and *E. faecalis* resistant to ampicillin were identified. Among Gram‐negative (GN) bacteria, resistance to third‐generation cephalosporins, trimethoprim‐sulfamethoxazole, aminoglycosides, and carbapenems was reported for *E. coli, Klebsiella*, and other *Enterobactera*les. Resistance to carbapenems, fluoroquinolones, aminoglycosides, and trimethoprim‐sulfamethoxazole was identified in *Acinetobacter* spp., whereas *P. aeruginosa* exhibited resistance to ceftazidime, carbapenems, aminoglycosides, piperacillin‐tazobactam, and fluoroquinolones. AMR was defined as an isolate being resistant to one or more of the antibiotics listed above. High AMR‐incidence countries comprised regions that include Southern and Eastern Europe, Asia, and Africa. Susceptibility to antifungals was not evaluated, and *Candida* species were excluded from the denominator when calculating the crude prevalence rate of AMR.

### 2.3 Acquisition of Reference Data

Antimicrobial susceptibility results were compared with existing data from the European Antimicrobial Resistance Surveillance Network (EARS‐Net) [8], Antibiotic Resistance and Prescribing in European Children (ARPEC project) [4], and Swedish Surveillance of Antibiotic Resistance (SVEBAR) [9].

Reference data from EARS‐Net were based on routine clinical antimicrobial susceptibility data of invasive isolates collected from local and clinical laboratories and reported to the European Centre for Disease Prevention and Control (ECDC) by appointed representatives from European Union (EU) member states. Surveillance data were readily accessible through the ECDC website and annual reports, and data from the 2018 report, “Surveillance of Antimicrobial Resistance in Europe,” were retrieved for this study [8]. ARPEC data included 19 hospitals across 12 European countries (Estonia, France, Germany, Greece, Italy, Lithuania, the Netherlands, Portugal, Slovenia, Spain, Switzerland, and the United Kingdom) in 2011–2012 [4]. The SVEBAR national IT system provided early alerts and continuous resistance monitoring by collecting susceptibility data for invasive isolates of selected pathogens from microbiological laboratories, with results transferred daily. These data were available on the Public Health Agency of Sweden website, and information from 2017 was retrieved for this study [7].

### 2.4 Statistical Analysis

Differences in the occurrence of AMR between HAI and CAI, between children before and after 1 year of life, and across different time periods were compared using the chi‐square test or Fisher's exact test in case of small sample size. Statistical significance was set at *p < 0*.05. Analyses were performed using test calculators in Social Science statistics (v1.0; https: www.ocscistatistics.com/tests/). Additionally, trends in acquired AMR occurrence over the study period for different paediatric risk groups were analysed using Kendall rank correlation (Wessa, 2017 and Kendall tau Rank Correlation (v1.0.13) in Free Statistics Software (v1.2.1; Office for Research Development and Education, https://www.wessa.net/rwasp_kendall.wasp/)).

This study was approved by the Regional Ethics Review Board (Reference Dnr. 2019–050119, 2013/2056–32, and 2009/1063–31/2).

## 3 Results

### 3.1 Trends of AMR 1998–2018

AMR occurrence was analysed in 1977 invasive isolates from paediatric patients with BSI during the study period. AMR was detected in 9.2% (181/1977) of isolates. The annual distribution of GP and GN isolates with acquired AMR is shown in Figures 1a,b and 2 demonstrating a significant increase in resistance among GP (*τ* = 0.61, *p < 0*.001) and GN (*τ* = 0.53, *p < 0*.01) isolates over time.

**FIGURE 1:**
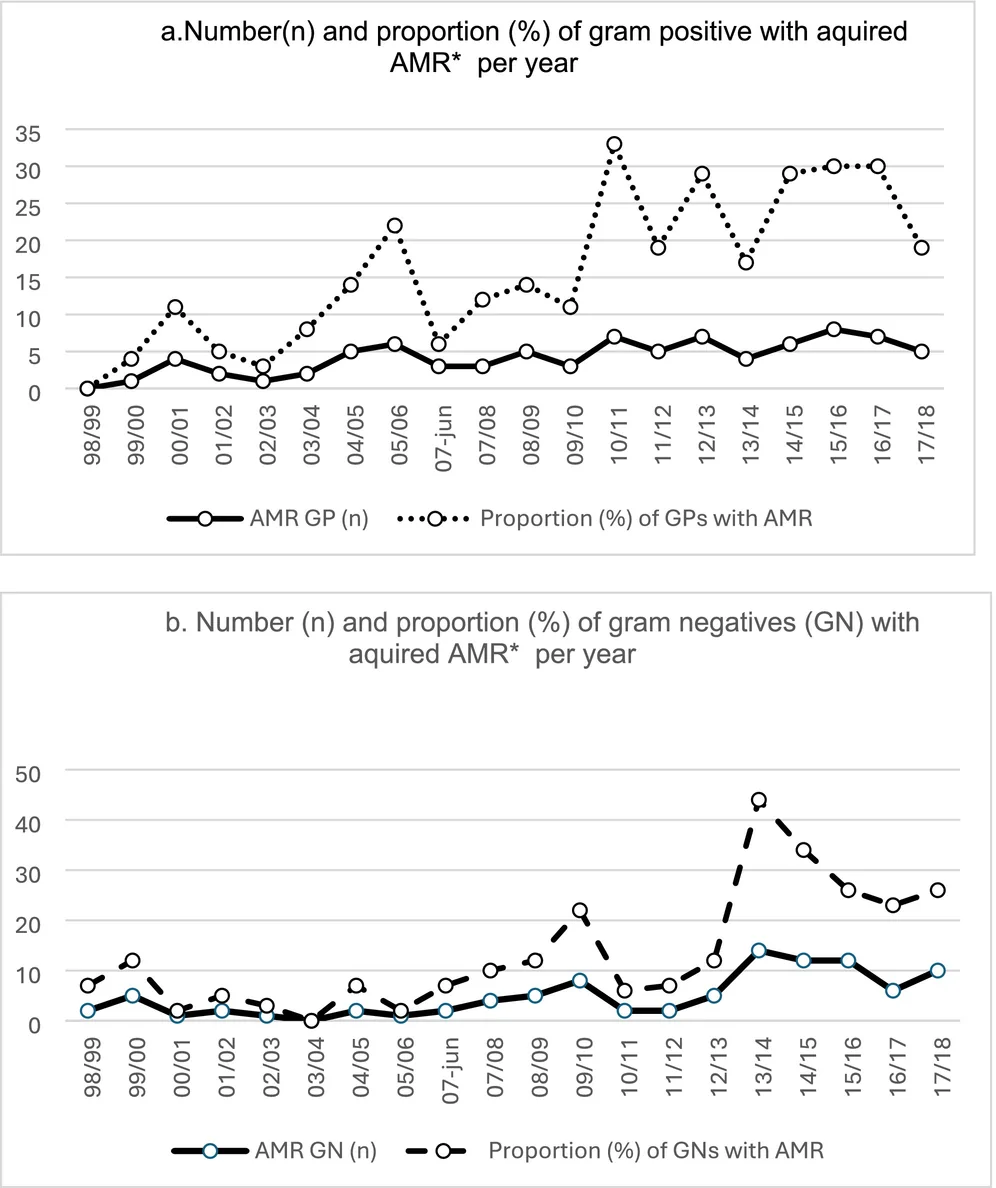
(a) Total number and proportion of antimicrobial‐resistant Gram‐positive, (b) and total number and proportion of Gram‐negative isolates obtained from children with bloodstream infections at Astrid Lindgren's Children's Hospital from 1998 to 2018.

**FIGURE 2:**
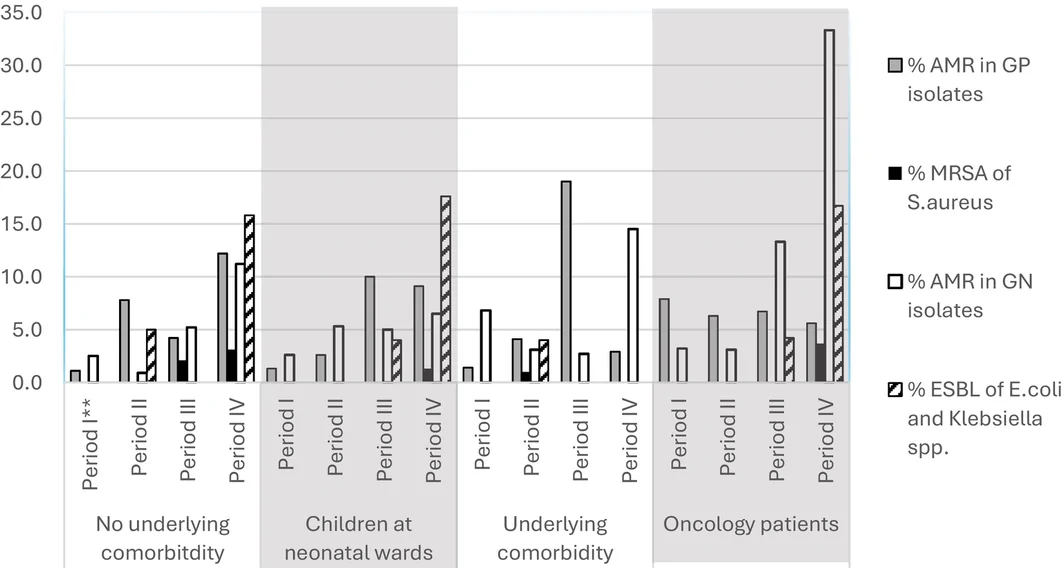
Proportion of AMR isolates among Gram‐positive bacteria (MRSA/*S.aureus*), Gram‐negative bacteria, and ESBL‐producing *E. coli* and *K. pneumoniae* , categorized by study period** and defined medical risk group. *The following Gram‐positive isolates were considered: *S. aureus* with resistance to methicillin and lincosamide, *S. pneumoniae* with resistance to macrolides and lincosamides, and penicillin non‐susceptibility. *S. agalactiae* resistance to macrolides. *Enterococcus faecium* resistance to vancomycin and *E. faecalis* resistance to ampicillin. The following Gram‐negative isolates were considered: *E. coli, Klebsiella*, and other *Enterobacterales* resistant to third‐generation cephalosporins, trimethoprim‐sulfamethoxazole, aminoglycosides, and carbapenems. *A. baumannii* resistant to carbapenems, fluoroquinolones, aminoglycosides, and trimethoprim‐sulfamethoxazole. *P. aeruginosa* resistant to ceftazidime, carbapenems, aminoglycosides, piperacillin‐tazobactam, and fluoroquinolones. **Period I: July 1998 to June 2003; period II: July 2003 to June 2008; period III: July 2008 to June 2013; and period IV: July 2013 to June 2018.

### 3.2 Gram‐Positive (GP) Isolates

Total GP isolates numbered 1243, and AMR was detected in 6.9% (86/1243) of GP isolates. During the last 5‐year period, *Staphylococcus aureus* resistant to lincosamides became more prevalent at 13.4% (7/52) compared to 1.2% (1/85) during the first 10 years of the study period (*p* < 0.01). Methicillin‐resistant *S. aureus* (MRSA) was found in eight *S. aureus* isolates during the study period, with six occurring in the final study period (2013–2018), resulting in an MRSA prevalence of 3.3% (6/179). Seventy‐five percent (6/8) of MRSA cases were CAI, with six children having a history of recent travel (*n* = 1), hospital admission (*n* = 1), or household members from regions with high AMR prevalence (*n* = 4). BSI caused by *Streptococcus pneumoniae* showed a non‐significant increase in resistance to macrolides, tetracycline, or trimethoprim‐sulfamethoxazole from 11.2% (13/116) before the introduction of the pneumococcal conjugate vaccine in 2007 to 17.7% (11/62) after 2008 (*p* = 0.22). However, penicillin non‐susceptible *S. pneumoniae* (PNSP) remained rare, with a prevalence of 1.6% (2/125) before and 1.9% (1/53) after 2007.

### 3.3 Gram‐Negative (GN) Isolates

Total GN isolates numbered 734 and AMR was detected in 12.9% (95/734) of GN isolates. AMR also increased in the last 5 years of the study for *E. coli* , *Salmonella* spp., *H. influenzae* , and *N. meningitidis* . Extended‐spectrum β‐lactamase‐producing Enterobacterales (ESBL) increased from 1.5% (2/130) in 1998–2008 to 14.1% (12/85) (*p* < 0.001), representing 15.5% (9/58) of *E. coli* and 11.1% (3/27) of *K. pneumoniae* isolates during the last 5 years of the study. Of the 17 patients with ESBL, seven were oncology patients, five were hospitalized in the NICU, and five were children without underlying diseases. Nine of 17 patients had no known history of antibiotic therapy; HAI accounted for 10 of these cases, and five children were hospitalized in a country with a high AMR incidence. Overall, 82.4% (14/17) of children with ESBL‐producing *Enterobacterales* had a history of recent travel (*n* = 4), hospital admission (*n* = 5), or household members from regions with high AMR prevalence (*n* = 9). Moreover, 64.7% (11/17) of ESBL isolates exhibited producing resistance to three or more antibiotic classes, meeting the criteria for multidrug resistance (MDR). Carbapenemase‐(ESBL_CARBA_) was found in one *K. pneumoniae* isolate, representing 0.1% (1/734) of GN isolates.

### 3.4 Occurrence of AMR in Different Risk Groups

Overall, the occurrence of AMR increased in all groups; however, the different risk groups exhibited different AMR patterns. The proportion of AMR isolates varied across risk groups, with rates of 9.5% (53/556) among children without underlying comorbidities, 7.8% (32/411) in neonates admitted to the NICU, 7.4% (41/552) in children with underlying comorbidities, and 12.0% (55/458) among oncology patients.

Among oncology patients, the increase in AMR was mainly observed in GN pathogens, accounting for 82.7% (24/29) of AMR isolates during the final study period.

Among children without underlying comorbidities, 98% (52/53) of BSI caused by AMR isolates were CAI, with GP isolates accounting for 58.5% (31/53) of cases.

In contrast, GN isolates were more prevalent in neonates admitted to the NICU (50.0%), children with underlying comorbidities (51.2%), and oncology patients (67.3%), where HAI represented 78.1%, 62.5%, and 54.5% of AMR cases, respectively.

Among children with BSI admitted to the paediatric intensive care unit (PICU), 9.3% (25/266) of isolates expressed AMR.

### 3.5 Age and Infection Type

Of the 1977 isolates, 915 (46.3%) were obtained from children younger than 1 year. The occurrence of AMR in invasive isolates from children younger and older than 1 year is presented in Table 1a, revealing a significantly higher AMR rate for *E. coli* in children aged > 1 year (*p* < 0.01) compared to infants aged < 1 year. Table 1b shows the distribution of AMR in isolates from children with CAI and HAI infections, showing that AMR was higher in *E. coli* isolates from HAI cases than from CAI cases, whereas no other significant differences were detected for other pathogens.

**TABLE 1a:** Occurrence of antimicrobial resistance (AMR) in invasive isolates among children aged < 1 and ≥ 1 year.

Pathogen	< 1 year		≥ 1 year	
	Total	With AMR	Total	With AMR
		*n* (%)		*n* (%)
*Staphylococcus aureus*	289	12 (4.2)	365	19 (5.2)
*Streptococcus pneumoniae*	46	6 (13.0)	132	18 (13.6)
*Enterococcus faecalis*	101	0	46	0
*Enterococcus faecium*	16	6 (37.5)	29	11 (37.9)
*Streptococcus agalactiae*	111	10 (9.0)	5	0
Other Gram‐positive bacteria	16	1 (6.3)	87	3 (3.4)
Gram‐positive bacteria total	579	35 (6.0)	664	51 (7.2)
*Escherichia coli*	141	24 (17.0)	75	23 (30.1)*
*Klebsiella pneumoniae*	37	6 (16.2)	42	11 (26.2)
Other *Enterobacter*ales	105	6 (5.7)	115	7 (6.0)
*Acinetobacter baumannii*	13	0	21	0
*Pseudomonas aeruginosa*	24	2 (8.3)	59	7 (11.9)
Other Gram‐negative bacteria	25	3 (12.0)	76	6 (7.9)
Gram‐negative bacteria total	346	41 (11.8)	388	54 (13.9)
All isolates	925	76 (7.7)	1052	105 (9.6)

* Significant at *p* < 0.01.

**TABLE 1b:** Antimicrobial resistance (AMR) in invasive isolates among children with community‐acquired infections (CAI) and healthcare‐associated infections (HAI).

Pathogen	HAI		CAI	
	With AMR		With AMR	
	Total	*n* (%)	Total	*n* (%)
*Staphylococcus aureus*	362	16 (4.4)	292	15 (5.1)
*Streptococcus pneumoniae*	9	1 (11.1)	169	23 (13.6)
*Enterococcus faecalis*	113		34	
*Enterococcus faecium*	36	14 (38.9)	9	3 (33.3)
*Streptococcus agalactiae*	17	2 (11.7)	99	8 (8.1)
Other Gram‐positive bacteria	24	2 (8.3)	79	2 (2.5)
Gram‐positive bacteria total	561	35 (6.2)	682	51 (7.5)
*Escherichia coli*	75	24 (32.0)	141	23 (16.3)*
*Klebsiella pneumoniae*	44	8 (18.2)	35	9 (25.7)
Other *Enterobacterales*	123	6 (4.8)	97	7 (7.2)
*Acinetobacter baumannii*	28		6	
*Pseudomonas aeruginosa*	58	9 (15.5)	26	
Other Gram‐negative bacteria	17	2 (11.7)	84	7 (8.3)
Gram‐negative bacteria total	345	49 (15.1)	389	46 (11.8)
All isolates	906	84 (9.2)	1071	97 (9.1)

* Significant at *p* < 0.01.

### 3.6 Clinical Outcomes

The crude mortality rate was 3.3% (6/181) for AMR‐related BSI compared to 5.9% (106/1796) for susceptible isolates.

### 3.7 Low AMR From an International Perspective

A comparison of AMR data from this study with findings from other paediatric surveillance programs (ARPEC data) and mixed adult‐paediatric datasets (ECDC and SVEDBAR) is presented in Table 2. The results showed significantly lower AMR rates in this study compared to European and ARPEC data, while mirroring national Swedish adult trends.

**TABLE 2:** Comparison of findings at Astrid Lindgren's Children's Hospital (ALCH) with paediatric surveillance data and resistance percentages from other surveillance sources that include both adult and paediatric data.

Pathogen and antibiotic class	Paediatric data	Mixed adult and paediatric data		
	ALCH 2013–2018	ARPEC (5) 2011–2012	EARS‐Net (3), European populations based, mean, 2018	EARS‐Net (3) SWE 2018 or SVEBAR (8)
*Staphylococcus aureus*				
Methicillin‐resistant	3.3	16.4	16.4	1.9
Lincosamides	8.4			3.4
*Enterococcus faecalis*				
High‐level gentamicin	0.0	30.5	27.1	14.7
*Enterococcus faecium*				
Vancomycin	0.0	8.3	17.3	1.4
*Streptococcus pneumoniae*				
PNSP	0.0	13.4	10.8	5.2
Macrolides	7.1	33.1	15.3	4.5
*Escherichia coli*				
3G cephalosporins	15.5	10.7	15.1	8.3
Aminoglycosides	5.2	13.5	11.1	7.7
Fluoroquinolones	15.5	8.5	25.3	18.1
Carbapenems	0.0	0.0	0.1	< 0.1
*Klebsiella pneumoniae*				
3G cephalosporins	11.1	29.9	31.7	5.5
Aminoglycosides	0.0	26.2	22.7	3
Fluoroquinolones	7.4	7.5	31.6	10.1
Carbapenems	0.0	1.9	7.5	0.2
*Pseudomonas aeruginosa*				
Pip‐tazobactams	8.7	36.0	18.3	7.8
Ceftazidime	4.4	25.8	14.1	6.1
Aminoglycosides	0.0	27.3	11.8	1.0
Fluoroquinolones	4.4	23.4	19.7	7.1
Carbapenems	13.1	32.8	17.2	4.4

### 3.8 Antimicrobial Prescribing During the Study Period

During the study period, the total antimicrobial consumption remained stable (*τ* = −0.02, *p* = 1) (Figure 3). Third‐generation cephalosporins were the most commonly prescribed agents with no declining trend (*τ* = 0.38, *p* = 0.015), while piperacillin‐tazobactam prescriptions increased, probably due to its recommendation for treating neutropenic fever.

**FIGURE 3:**
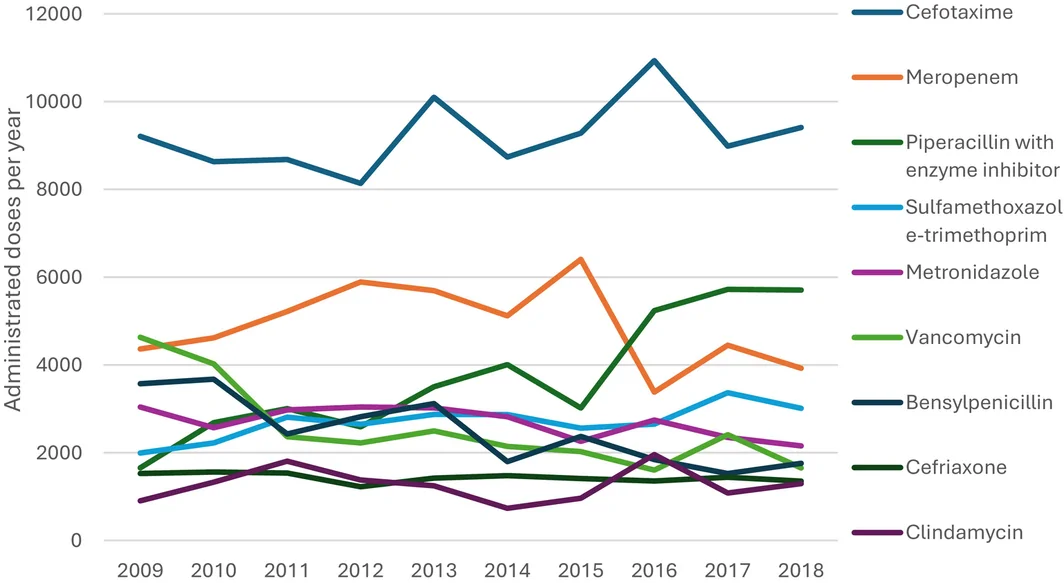
Most frequently used antimicrobial drugs at Astrid Lindgren Children's Hospital from 2008 to 2018, measured by total administered doses per year.

## 4 Discussion

Three significant changes emerged from this study. First, an increase in AMR occurrence was observed. Second, total antibiotic consumption among hospitalized patients remained unchanged throughout the study period. Third, a strong association was identified between the occurrence of ESBL and MRSA and a history of recent travel, prior hospital admission of the patient, or household members with similar exposure.

The observed increase in AMR was mainly due to AMR in GN bacteria. The proportion of ESBL‐producing strains among BSI‐causing *Enterobacterales* increased from approximately 1.5% during the first decade to 14.1% in the final study years, exceeding the reported proportion in Swedish adult data. Notably, a concurrent increase in MDR *E. coli* ESBL was observed, with 64.7% of ESBL isolates exhibiting MDR characteristics in the last 5 years. Although studies on Swedish adults with ESBL‐BSI have reported similar or even higher MDR rates, the high MDR prevalence in invasive isolates contrasts with that observed in ESBL carriers, where MDR is less frequent [10]. A history of travel for the child or a household member was evident in 82.4% of ESBL cases, reinforcing the link between international exposure and AMR acquisition.

AMR also increased in GP bacteria, primarily due to rising *S. aureus* resistance to lincosamides. MRSA prevalence reached an estimated 3% of *S. aureus* BSI in the last 5 years, although the low number of cases warrants cautious interpretation of trends. While the observed MRSA rate remains low compared to European and global data, it is higher than previously reported in Swedish datasets [4, 11, 12, 13].

Our study did not reveal PNSP, which contrasts with reported non‐susceptibility rates of 5.5%–13.5% from ARPEC [4] and Swedish EARS‐Net data.

Oncology patients exhibited the highest prevalence of both BSI and AMR isolates, with the AMR rate increasing from 6% in the first decade to 22% in the last 5 years period. Notably, 17% of *E. coli* and *Klebsiella* spp. isolates in this group were ESBL‐producing. Despite this increase, no association was found between AMR and a higher risk of infection‐related mortality in patients with oncology, differing from the findings of Levene et al. [14].

During the study period, a NICU outbreak of an ESBL‐producing MDR *Klebsiella pneumoniae* strain resulted in one BSI with a fatal outcome and two additional fatalities, both in patients without positive blood cultures. Fourteen children were colonized and did not develop infections [15]. To our knowledge, no other outbreaks or secondary cases of ESBL or MRSA were detected in our hospital. NICUs are recognized as high‐risk environments for HAI, particularly in low‐ and lower‐middle‐income countries [16, 17]. However, our data indicate a low incidence of BSI caused by *Acinetobacter baumannii* in both NICU and PICU settings [18], aligning with national AMR surveillance data in paediatric HAIs from the US [19]. The ARPEC project reported a higher resistance percentage in children over 1 year than in infants under 1 year [4]. In contrast, our study found this difference only for *E. coli* . Additionally, we did not observe the high resistance of *E. coli* and *K. pneumoniae* to aminoglycosides or *P. aeruginosa* to carbapenems, as reported by ARPEC.

Moreover, AMR in *E. coli* was more prevalent among children with HAI than those with CAI. Although pathogen distribution varied between the CAI and HAI cases, these differences were primarily explained by the presence of comorbidities rather than the classification of the infection as CAI or HAI. The timing of positive blood samples does not differentiate pathogens causing HAI from CAI BSI, as reported in the UK by Henderson et al. [20].

The lower prevalence of AMR among children with underlying comorbidities compared to those without is an intriguing finding. Children with comorbidities typically experience more frequent hospitalizations and previous antibiotic treatments, both of which are known risk factors for AMR. Hypothetically, this observation could be explained by reduced travel within this group. However, it also highlights a limitation of the study: we did not compare prior hospitalizations or international travel among children without detected AMR. Another limitation is that we were unable to compare AMR prevalence within the same time periods, which may have contributed to differences in the results.

We did not observe any differences in mortality between patients with BSI caused by AMR isolates and isolates susceptible to empiric antibiotics, which contrasts with other countries with high AMR prevalence [21, 22, 23]. However, our results align with reports from regions with lower AMR prevalence [24]. The overall low mortality rates of both BSI and AMR could partly explain the absence of a negative effect of AMR on mortality.

To further reduce AMR, global efforts to target antibiotic overuse are required. However, additional preventive strategies may also affect AMR. Messina et al. found that pneumococcal vaccination significantly decreased the risk of antimicrobial‐resistant *S. pneumoniae* carriage, emphasizing the need for pathogen‐specific interventions [25]. Universal coverage with a pneumococcal conjugate vaccine was estimated to reduce antibiotic use for *S. pneumoniae* ‐related pneumonia by 47% [26]. Klugman and Black highlighted vaccination as a crucial tool for reducing antimicrobial use and AMR, advocating for the development of new vaccines as an effective prevention strategy [27]. Although stewardship programs targeting a single antibiotic class have shown limited impact on AMR, combining stewardship initiatives with hand hygiene interventions has led to AMR reduction in adults [28].

In conclusion, an increase in AMR was observed, particularly in ESBL‐producing *Enterobacterales*, despite stable prescription rates of third‐generation cephalosporins. The risk factors for AMR remain complex; however, recent travel or hospital admissions—either for the child or household members—may contribute to the observed trends.

Moreover, most children hospitalized in Sweden or other low‐AMR prevalence settings do not need empirical antibiotic coverage for ESBL‐producing *Enterobacterales* or MRSA. However, such coverage should be considered for children with a history of travel or hospitalization in countries with high AMR prevalence, those with household members who have travelled to or been hospitalized in such countries, and those previously known to carry resistant bacteria. The observed temporal changes underscore the importance of continuous BSI and AMR surveillance to inform empirical antibiotic treatment and strengthen antimicrobial stewardship.

## Author Contributions

**Per Nydert:** data curation, writing – review and editing. **Rutger Bennet:** investigation, conceptualization, writing – review and editing, data curation, formal analysis, validation. **Margareta Eriksson:** conceptualization, investigation, methodology, data curation, supervision. **Joachim Luthander:** conceptualization, writing – original draft, funding acquisition, investigation, data curation, visualization, formal analysis, writing – review and editing, methodology, validation. **Christian G. Giske:** data curation, writing – review and editing, supervision. **Anna Nilsson:** supervision, writing – review and editing, conceptualization, funding acquisition, investigation, methodology, writing – original draft, validation.

## Funding

This work was supported by Stiftelsen Sven Jerrings Fond. VINNOVA, 2018‐03340, 2021‐02699. Swedish Cancer Foundation.

## Conflicts of Interest

The authors declare no conflicts of interest.

## Data Availability

The data that support the findings of this study are available from the corresponding author upon reasonable request.
